# Significance of a PTEN Mutational Status-Associated Gene Signature in the Progression and Prognosis of Endometrial Carcinoma

**DOI:** 10.1155/2022/5130648

**Published:** 2022-02-23

**Authors:** Ying Wu, Jun Wang, Lina Ge, Qing Hu

**Affiliations:** Department of Obstetrics and Gynecology, Shengjing Hospital of China Medical University, Shenyang, China

## Abstract

**Background:**

PTEN mutations have been reported to be involved in the development and prognosis of endometrial carcinoma (EC). However, a prognostic gene signature associated with PTEN mutational status has not yet been developed. In this study, we generated a PTEN mutation-associated prognostic gene signature for EC.

**Methods:**

We obtained the single-nucleotide variation and transcriptomic profiling data from The Cancer Genome Atlas database as training data and implemented the least absolute shrinkage and selection operator (LASSO) Cox regression algorithm to establish a PTEN mutation-associated prognostic gene signature. The overall survival rates of the high-risk and low-risk groups were determined with the Kaplan-Meier (K-M) method, and the accuracy of risk score prediction was tested by using the receiver operating characteristic (ROC) curve.

**Results:**

The K-M curves revealed that the EC patients with PTEN mutations augured favorable survival outcomes. Differential expression analysis between the EC patients with PTEN mutation and wild-type PTEN identified 224 differentially expressed genes (DEGs). Eighty-four DEGs that manifested prognostic value were fitted into the LASSO-Cox analysis, and a PTEN gene signature with seven mutation-associated prognostic genes that showed robust prognostic ability was constructed; this signature was then successfully validated in the other two datasets from the cBioPortal database as well as with 60 clinical specimens. Furthermore, the PTEN mutation-associated prognostic gene signature proved to be an independent prognostic predictor of EC. Remarkably, the EC patients in the high-risk group were characterized by higher tumor stages and grades as well as lower tumor mutation burden with respect to EC, with a poor survival outcome. Collectively, the PTEN mutation-associated prognostic gene signature that we developed could now be used as a favorable prognostic biomarker for EC.

**Conclusion:**

In summary, we developed and validated a prognostic predictor for EC associated with PTEN mutational status that may be used as a favorable prognostic biomarker and therapeutic target for EC.

## 1. Background

Endometrial carcinoma (EC) is a common malignancy of the female reproductive system, and its incidence is increasing [1]. EC is a heterogeneous tumor, and the prognosis of patients is closely related to tumor grade and stage; early, accurate, and effective diagnosis is thus helpful in improving the prognosis of the EC patients [2]. Surgery and postoperative radiotherapy are routinely used methods for treating this condition, but there is still a lack of effective treatment for recurrent or progressive EC [3]. Therefore, there is an urgent need to identify additional biological markers for the prognostic prediction of EC.

Phosphatase and tensin homolog on chromosome 10q23 (PTEN), a recognized tumor suppressor gene, is one of the most common mutated genes in human tumors, and it can be detected in a variety of tumor tissues, including EC [4]. Kong et al. [5] reported that the mutation rate of PTEN in EC was higher relative to other tumors and that 37%–61% of their EC patients manifested a PTEN gene mutation. Investigators [6] have also demonstrated that PTEN-deficient endometrial epithelial cells were more likely to convert to complex atypical hyperplasia in response to estrogen stimulation and thus develop into EC; and, accordingly, PTEN deficiency is generally considered to be an early event in EC development. Another study [7] also confirmed that functional loss of PTEN was an early cancerous event that exhibited a higher frequency of PTEN mutations in precancerous or stage I tumors than in advanced or even metastatic EC and that PTEN mutations were associated with more favorable outcomes. Thus, PTEN mutations in endometrial hyperplasia may serve as an early warning indicator for increased cancer risk [8].

In view of the important role played by PTEN mutations in the progression and prognosis of EC, we herein fully revealed the mutational landscape of PTEN in EC and developed a PTEN mutational status-associated prognostic gene signature to predict the prognosis of EC based on The Cancer Genome Atlas (TCGA) database. We also executed external validation using two other datasets from the cBioPortal database as well as 60 clinical specimens so as to prove prognostic ability. Furthermore, we demonstrated the relationships between the PTEN mutational signature and stage/grade as well as tumor mutation burden (TMB) in EC. We posit that the PTEN mutation-associated prognostic gene signature can be used as an acceptable prognostic biomarker for EC.

## 2. Materials and Methods

### 2.1. Data Acquisition

**Figure 1:**
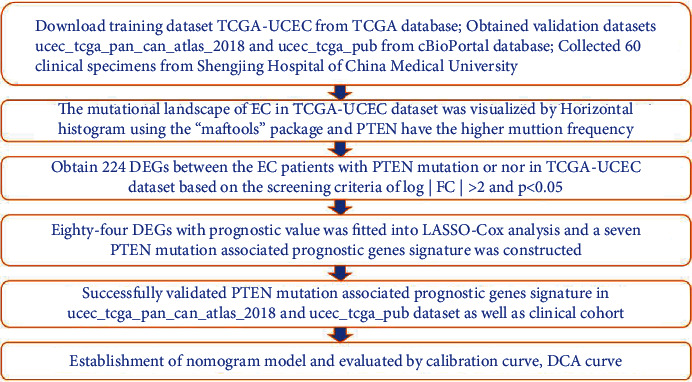
Study workflow. Abbreviations are defined as follows: TCGA: The Cancer Genome Atlas; DEGs: differentially expressed genes; LASSO: least absolute shrinkage and selection operator; UCEC: uterine corpus endometrial carcinoma; DCA: decision curve analysis.

Single-nucleotide variation, transcriptomic profiling datasets, and corresponding clinical information of 529 EC patients were downloaded from the TCGA database (https://portal.gdc.cancer.gov/) and considered as the training dataset. The transcriptomic profiling datasets and corresponding clinical information of the validation datasets ucec_tcga_pan_can_atlas_2018 and ucec_tcga_pub included 527 and 331 EC patients, respectively, and were obtained from the cBioPortal database (http://www.cbioportal.org/study/summary?id=ucec_tcga). The clinical information included age, BMI, tumor stage and grade, overall survival time, and survival status (the flowchart of our study is shown in Figure 1).

### 2.2. Specimen Collection

We selected a total of 60 patients with primary endometrial cancer who were admitted to the Department of Gynecology and Obstetrics of Shengjing Hospital between January of 2016 and January of 2017. The patients in the primary endometrial cancer group were 35–60 years of age, with a mean age of 54.5 years. There were 30 cases of patients with FIGO stage I, 15 with stage II, and 15 with stages III–IV. There were 15 patients graded G1, 17 graded G2, and 28 graded G3. All patients underwent staging surgery for endometrial cancer, with removal of pelvic lymph nodes and abdominal para-aortic lymph nodes. A patient was confirmed as having endometrial cancer by pathologists, and none of the patients had received chemotherapy or radiotherapy prior to surgery. This study was approved by the Ethics Committee of Shengjing Hospital of the China Medical University, and informed consent was obtained from all patients. In addition, all methods were performed in accordance with the relevant guidelines and regulations.

### 2.3. Identification of Differentially Expressed Genes

We employed the “limma” package to identify the differentially expressed genes (DEGs) between the EC patients with PTEN mutation and those with wild-type PTEN. Our screening criteria for DEGs were log | FC | >2 and *P* < 0.05, and the results were visualized as heatmaps and volcano maps.

### 2.4. Construction and Validation of a PTEN Mutational Status-Associated Prognostic Signature

The “survival” package was used to screen the DEGs with prognostic value by univariate Cox regression analysis based on the screening criterion of *P* < 0.05. Next, the key DEGs with prognostic value were further selected by least absolute shrinkage and selection operator (LASSO) regression and stepwise regression analyses. We used multivariate Cox regression to calculate the regression coefficients of the key DEGs with prognostic value and generated a PTEN mutational status-associated prognostic signature. The risk score for each EC patient was calculated using the following formula: risk score = exp1 × *β*1 + exp2 × *β*2 ⋯ +expn × *β*n (expn represents the expression value of each key DEG with prognostic value, and *β*n represents the regression coefficient) [9]. The EC patients were classified into the high- and low-risk groups based on the median risk score, and the Kaplan-Meier (K-M) method and log-rank test were applied to evaluate the survival between the high- and low-risk groups. Time-dependent and time-independent receiver operating characteristic (ROC) curves were constructed to evaluate the prognostic ability of the PTEN mutational status-associated prognostic signature, and we validated the signature using the ucec_tcga_pan_can_atlas_2018 and ucec_tcga_pub. Finally, the univariate and multivariate Cox regression analyses were used to determine whether the PTEN-associated signature possessed independent prognostic value in both testing and validation datasets.

### 2.5. Real-Time qPCR

Real-time qPCR was used to detect the relative expression levels of PTEN-associated genes in 60 EC tissues. Total RNA from EC samples was extracted by using TRIzol reagent (Invitrogen, USA) and reverse-transcribed to cDNA, and the RT-qPCR was performed using SYBR Premix Ex Taq (Takara, Japan). GAPDH was selected as an internal reference to detect the relative expression levels of PTEN-associated genes in EC tissues based on the 2^-*ΔΔ*Ct^ method, and the primer sequences for RT-qPCR are presented in Supplementary Table 1. Next, we established a PTEN-associated signature based on the relative expression levels of PTEN-associated genes to verify the results of our bioinformatics analysis. A K-M curve was used to evaluate the survival between the high- and low-risk groups, and we constructed ROC curves to evaluate the prognostic ability of the PTEN-associated signature.

### 2.6. Construction and Validation of a Nomogram Model Based on the PTEN-Associated Signature and Clinical Factors

The “rms” and “survival” packages were used to construct a nomogram model based on the PTEN-associated signature and clinical factors, and consistency between actual and predicted survival rates was assessed with calibration curves. We employed decision curve analysis (DCA) to evaluate the accuracy of the prognostic prediction model [10, 11].

### 2.7. Statistical Analysis

We used the “maftools” package to reveal PTEN mutational status in EC. Perl was used to calculate the TMB of the patients with EC from TCGA database, and the Wilcoxon rank-sum test was employed for comparative analysis between the two groups. The DEGs related to overall survival (OS) were screened out by univariate Cox regression analysis, and the LASSO-Cox regression algorithm was used to establish the risk-prognosis model. The OS rates of the high-risk and low-risk groups were determined by the K-M method, and the accuracy of risk score prediction was assessed by the ROC curve. The univariate and multivariate Cox regression analyses were used to assess whether the PTEN-associated signature displayed independent prognostic value. The above statistics were analyzed with R (version 3.6.3) software, and a test level of *P* < 0.05 was considered statistically significant.

## 3. Results

### 3.1. PTEN Mutational Status in Endometrial Carcinoma

**Figure 2:**
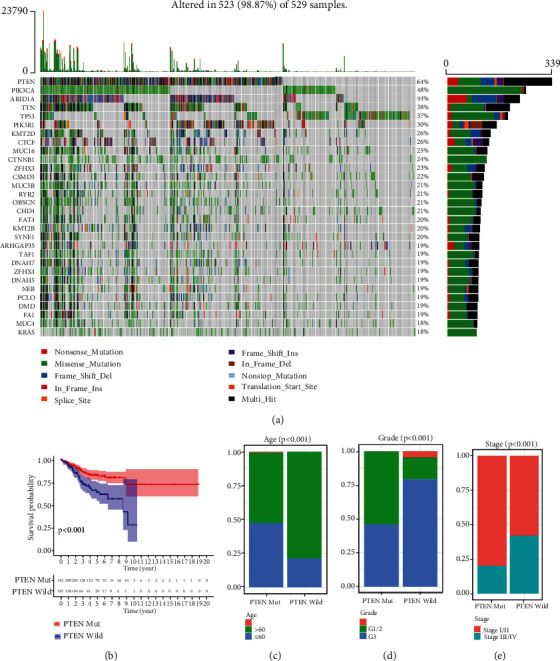
PTEN mutational status of endometrial carcinoma in The Cancer Genome Atlas (TCGA) dataset. (a) Mutational landscape of endometrial carcinoma (EC) in TCGA dataset. (b) Kaplan-Meier curves revealed that the EC patients with PTEN mutation showed a favorable survival outcome. (c–e) Percentage chart showing that the PTEN mutation occurred more frequently at a younger age and with a lower tumor stage and grade in EC patients.

The mutational landscape of EC in TCGA database was visualized by horizontal histogram using the “maftools” package, and PTEN depicted a high mutation frequency (64%; Figure 2(a)). The K-M curves revealed that the EC patients with PTEN mutation (PTEN mut) exhibited a longer survival time than the patients with wild-type PTEN (PTEN wild; *P* < 0.001; Figure 2(b)), and the percentage chart shows that the PTEN mutation occurred more frequently in the younger patients with a lower stage and grade of cancer (*P* < 0.001; Figures 2(c)–2(e)).

### 3.2. Identification of Differentially Expressed Genes and Construction of the PTEN Mutational Status-Associated Prognostic Signature

**Figure 3:**
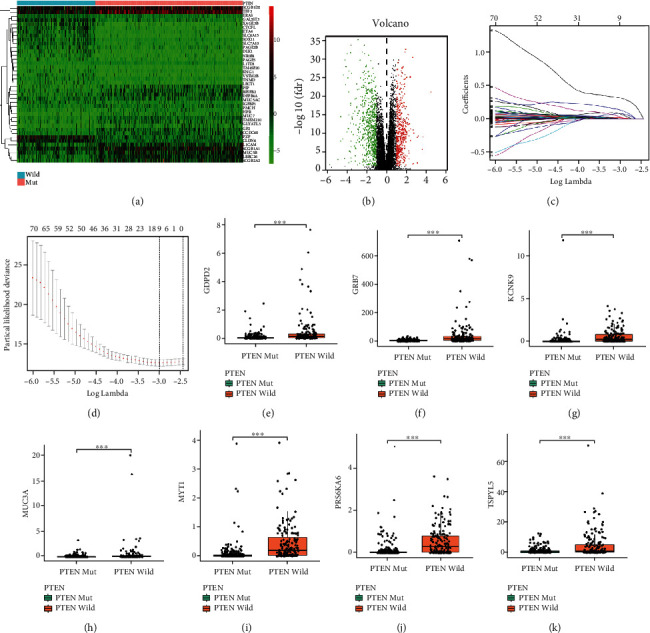
Identification of differentially expressed genes (DEGs) and construction of the PTEN mutational status-associated prognostic signature. (a) Heatmap showing the top 40 DEGs between the EC patients with and without PTEN mutation. (b) Volcano plot showing the DEGs between the EC patients with and without PTEN mutation. (c) LASSO coefficient profiles of the 84 DEGs with prognostic value. (d) Nine PTEN mutation prognostic genes obtained from LASSO regression based on 10-fold cross-validation and minimal criteria. (e–k) The relative expression levels of the seven prognostic genes (GDPD2, GRB7, KCNK9, MUC3A, MYT1, RPS6KA6, and TSPYL5) between the EC patients with and without PTEN mutation.

Considering the robust prognostic value of PTEN mutational status, we developed a PTEN mutational status-associated prognostic signature to predict the prognosis of EC. First, the “limma” package was used to identify 224 DEGs (37 upregulated genes and 187 downregulated genes) between the EC patients with PTEN mutation and those without, based on the screening criteria of log | FC | >2 and *P* < 0.05 (Figures 3(a) and 3(b); Supplementary Table 2). Eighty-four DEGs with prognostic value were selected using univariate Cox regression analysis based on the screening criterion of *P* < 0.05 (Supplementary Table 3). We performed LASSO analysis based on the 84 DEGs and obtained nine genes (Figures 3(c) and 3(d)). Stepwise regression analyses then further reduced the nine genes to seven, and a PTEN mutation-associated prognostic signature was constructed. The risk score for each EC patient was calculated using the following formula: risk score = 0.2023 × expGDPD2 + 0.0029 × expGRB7 + 0.2875 × expKCNK9 + 0.1244 × expMUC3A + 0.4495 × expMYT1 + 0.2650 × expRPS6KA6 + 0.0359 × expTSPYL5 (Table 1). Differential analysis revealed that all seven genes were more highly expressed in the PTEN-wild group than in the PTEN-mut group (Figures 3(e)–3(k)).

### 3.3. Evaluation and Validation of the PTEN Mutational Status-Associated Prognostic Signature

**Figure 4:**
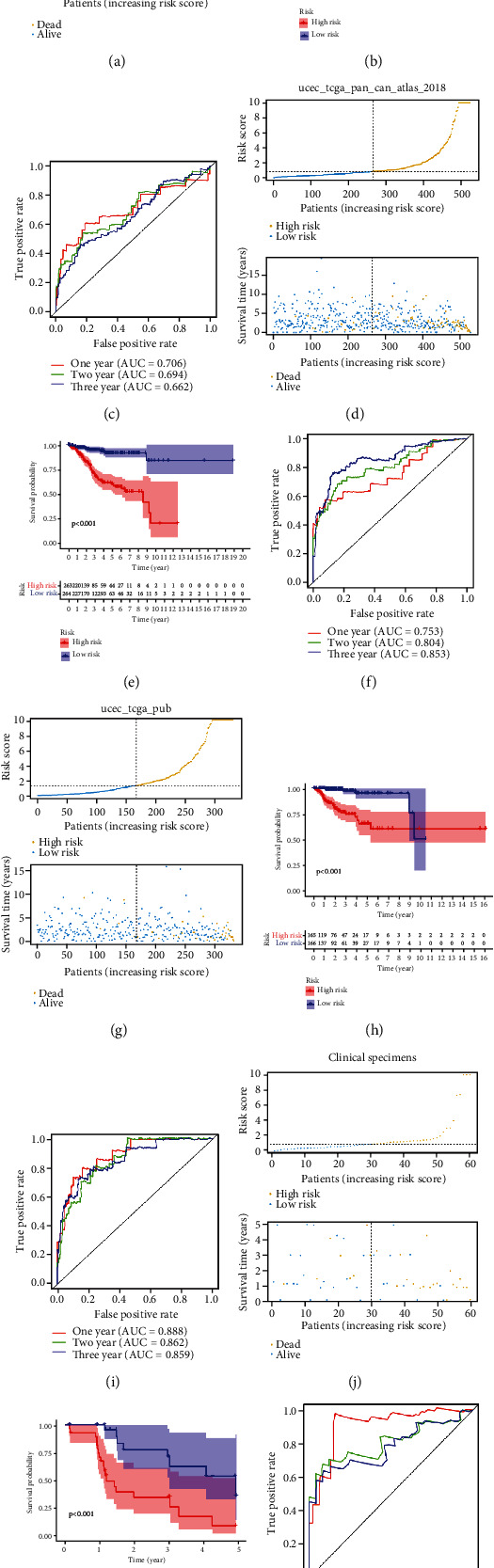
Evaluation and validation of the PTEN mutational status-associated prognostic signature in the training and validation datasets. Risk score, survival status, K–M curve, and ROC curve in TCGA (a–c), ucec_tcga_pan_can_atlas_2018 (d–f), ucec_tcga_pub dataset (g–i), and clinical specimens (j–l).

We then evaluated and validated the prognostic ability of the PTEN mutational status-associated prognostic signature in both the training and validation datasets. The risk score and survival status of the EC patients are shown in Figures 4(a), 4(d), 4(g), and 4(j); and the EC patients were classified into high- and low-risk groups based on the median risk score. The K-M curve analysis indicated that the patients in the high-risk group evinced a poor survival outcome (Figures 4(b), 4(e), 4(h), and 4(k)), and the values for the areas under the ROC curve (AUC) at 1, 3, and 5 years in TCGA dataset were 0.706, 0.694, and 0.662, respectively (Figure 4(c)). The AUC values at 1, 3, and 5 years in the ucec_tcga_pan_can_atlas_2018 dataset were 0.753, 0.804, and 0.853, respectively (Figure 4(f)); the respective values at 1, 3, and 5 years in the ucec_tcga_pub dataset were 0.888, 0.862, and 0.859 (Figure 4(i)); and the AUC values at 1, 3, and 5 years in our clinical specimens were 0.910, 0.806, and 0.782, respectively (Figure 4(l)). All of the above results highlighted the robust predictive potential of our PTEN mutational status-associated prognostic signature.

### 3.4. Independent Prognostic Value of the PTEN Mutational Status-Associated Prognostic Signature and Its Relationship with Clinicopathological Characteristics

**Figure 5:**
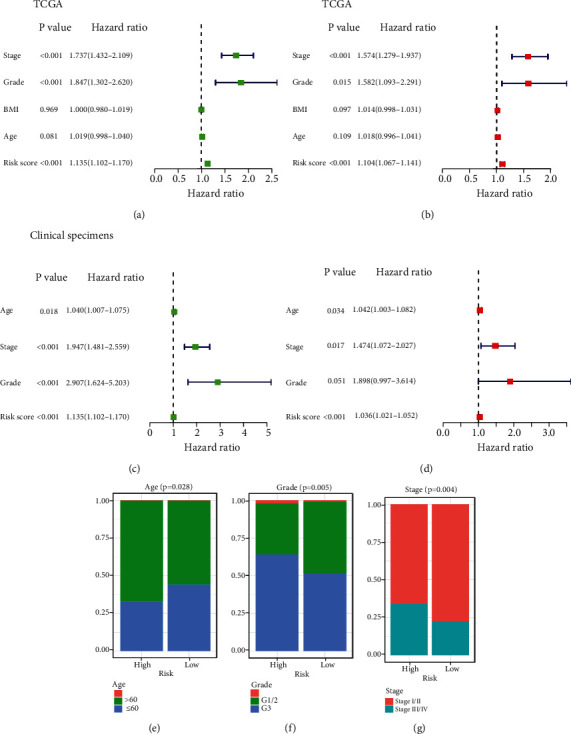
Independent prognostic value of the PTEN mutational status-associated prognostic signature and its relationship to clinicopathological characteristics. (a) Univariate Cox regression analysis of TCGA dataset. (b) Multivariate Cox regression analysis of TCGA dataset. (c) Univariate Cox regression analysis in the clinical cohort. (d) Multivariate Cox regression analysis in the clinical cohort. (e–g) Percentage chart showing that high-risk was associated with younger age and higher tumor stage and grade in EC patients.

To determine whether the PTEN-associated signature possessed independent prognostic value, the univariate and multivariate Cox regression analyses were performed in TCGA dataset and the clinical cohort, and both indicated that stage, grade, and risk score were related to the prognosis of the EC patients in TCGA dataset (Figures 5(a) and 5(b)). The univariate Cox regression analysis revealed that age, tumor stage and grade, and risk score were related to the prognosis of the EC patients in the clinical cohort, while the multivariate Cox regression analysis suggested that age, stage, and risk score were related to the prognosis of the EC patients (Figures 5(c) and 5(d)). Finally, in TCGA dataset, the percentage chart revealed that younger age and higher stage and grade of tumors in the EC patients were associated with high risk for EC (*P* < 0.05; Figures 5(e)–5(g)).

### 3.5. Construction and Validation of a Nomogram Model Based on the PTEN-Associated Signature and Clinical Factors

**Figure 6:**
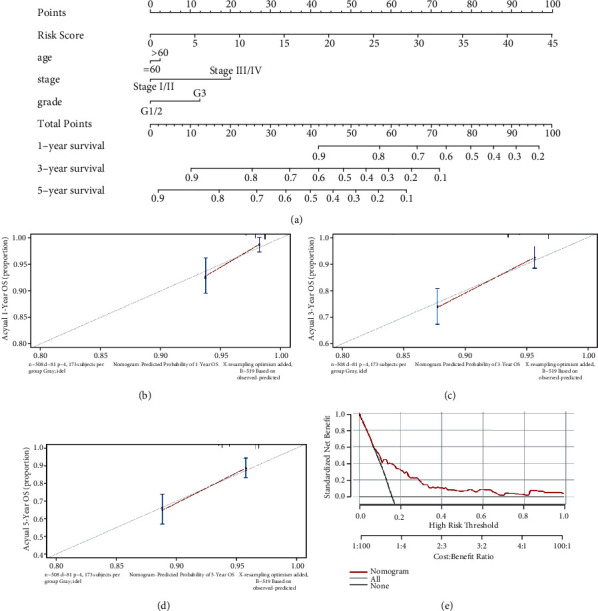
Construction of a nomogram model in TCGA dataset. (a) A nomogram for predicting the 1-, 3-, and 5-year overall survival rates of EC patients. (b–d) The calibration curve at 1, 3, and 5 years. (e) A DCA curve was used to evaluate the accuracy of the nomogram model.

The “rms” and “survival” packages were employed to construct a nomogram model based on the seven genes of the PTEN-associated signature and the clinical factors to predict the survival rates of EC patients at 1, 3, and 5 years (Figure 6(a)). The calibration curves for these periods revealed high consistency between the actual and predicted survival rates, suggesting the powerful predictive performance of the nomogram model (Figures 6(b)–6(d)); and the DCA curve indicated that the prognostic ability of the model was accurate (Figure 6(e)). Thus, we successfully validated our nomogram model in a clinical cohort (Supplementary Figure 1).

### 3.6. Mutational Landscape Associated with the PTEN Mutational Status-Associated Prognostic Signature

**Figure 7:**
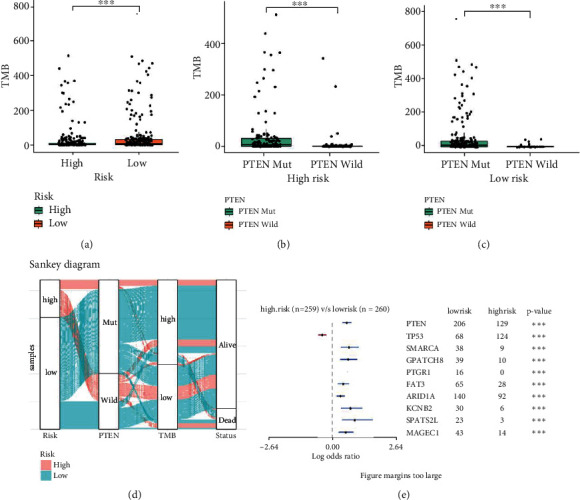
Mutational landscape associated with the PTEN mutational status-associated prognostic signature. (a) Differences in tumor mutation burden (TMB) between high- and low-risk groups. (b) Differences in TMB between patients with and without PTEN mutation in the high-risk group. (c) Differences in TMB between patients with and without PTEN mutation in the low-risk group. (d) Sankey diagram shows the relationships among risk score, PTEN mutational status, TMB, and survival status. (e) Mutational landscape associated with the PTEN-associated signature.

TMB refers to the total number of replacement and insertion/deletion mutations in each group of nucleobases in the coding region of the evaluated gene exon within the tumor cell genome [12]. Figures 7(a)–7(c) show that the patients in the high-risk group and those with the PTEN mutation reflected a higher TMB value, while the Sankey diagram shows the relationships among risk score, PTEN mutational status, TMB, and survival status (Figure 7(d)). Finally, we investigated the mutational landscape associated with the PTEN mutational status-associated prognostic signature and observed a higher PTEN mutation frequency in the high-risk group (Figure 7(e)).

## 4. Discussion

Mutations in the tumor suppressor PTEN constitute the most frequent type of mutation observed in EC [13], and PTEN has been shown to interact with cell adhesion complexes and to stabilize cell junctions, thereby reducing invasion and metastasis of a range of cancer cells that include EC [14–16]. Previous studies have scrutinized the influence of PTEN mutation on the progression and prognosis of endometrial cancer, but only few have focused on the development of a PTEN mutational status-associated prognostic signature [17–19]. In the present study, based on our analysis of the downloaded single-nucleotide variation and transcriptomic profiling datasets, we found that PTEN embodied a higher mutation frequency in EC patients and that PTEN mutation was associated with younger age and a lower stage and grade of tumors, as well as a favorable survival outcome for EC patients. We then explored and verified a PTEN mutational status-associated prognostic signature that was associated with the malignant progression and prognosis of EC patients. We posit that this PTEN mutation-associated signature constitutes a novel means to predict prognosis and to evaluate efficacy in EC and may thus become a new target for the treatment of EC patients in the future.

In this study, we developed a PTEN mutation-associated signature that included seven genes (GDPD2, GRB7, KCNK9, MUC3A, MYT1, RPS6KA6, and TSPYL5) that were selected by LASSO-Cox analysis, and by reviewing previous studies, we found that RPS6KA6 and TSPYL5 had been reported to be associated with the occurrence and development of EC. Ribosomal S6 kinase 4 (RSK4) is a tumor suppressor gene product (also known as RPS6KA6) that has been shown to be significantly downregulated in multiple malignancies—including those of the breast, colon, kidney, ovarian, and acute myeloid leukemia [20–27]; and it can inhibit tumor cell proliferation, invasion, and the epithelial mesenchymal transformation [28, 29]. One study [30] showed that hypermethylation of RSK4 in EC resulted in a lowered expression level of RSK4 in EC relative to normal endometrial tissues and that reduced RSK4 methylation was associated with higher EC grade. The testis-specific protein Y-encoded-like 5 (TSPYL5) is a member of the TSPYL family, and according to the current studies, the TSPYL5 expression is deleted or downregulated in many tumors [31]. As a new tumor suppressor molecule, the TSPYL5 is closely related to the malignant progression and prognosis of tumors [32–34] and has been reported to be associated with tumor differentiation, cell cycle, and survival in EC [35, 36]. Although the roles of GRB7, KCNK9, MUC3A, and MYT1 in EC have not been exposed, their actions in other tumors have been investigated. For example, growth factor receptor-bound protein 7 (GRB7) is an important bridging protein that is involved in the physiological and pathological processes such as embryonic development, angiogenesis, metabolic regulation, and tumorigenesis by binding to tyrosine kinase receptors (RTKs). GRB7 has also been described as being involved in cellular proliferation, migration, and invasion, cancer prognosis, and tumor-associated angiogenesis of a variety of tumors [37–39]. TASK-3 (also called KCNK9) is a member of the K2P potassium channel family, is overexpressed in a variety of tumor tissues such as breast cancer, gastric adenocarcinoma, ovarian cancer, and lung adenocarcinoma, and is closely related to tumor progression [40–43]. The MUC3A gene is mapped to a mucin cluster located on chromosome 7q22, is a tumor suppressor gene found to be expressed at low levels in a variety of tumors, and is involved in the malignant progression of tumors and in their prognoses [44, 45]. Myelin transcription factor 1 (MyT1) is principally expressed in developing central nervous system cells and mediates the proliferation and differentiation of oligodendrocytes and the formation of the myelin sheath of nerve cells [46]. Recent studies have shown that MYT1 is also involved in the malignant progression of gastric cancer, liver cancer, and glioblastomas [47–49]. Although glycerophosphodiester phosphodiesterase 2 (GDPD2) is primarily involved in lipid metabolism, its actions in EC and other tumors remain unreported [50].

To further evaluate the prognostic ability of the PTEN-associated signature in both training and validation datasets as well as in clinical specimens, an ROC curve was plotted and the AUC was calculated. We found a mean AUC of over 0.78, suggesting that the prognostic ability of the PTEN-associated signature was robust. Moreover, univariate and multivariate Cox regression analyses revealed that the PTEN-associated signature was an independent prognostic predictor for EC. Therefore, we posit that the PTEN-associated signature has the potential to be a promising clinical prognostic tool for EC.

## 5. Conclusions

In summary, we developed and validated a prognostic predictor for EC associated with PTEN mutational status. The PTEN mutation-associated prognostic gene signature may therefore be used as a set of favorable prognostic biomarkers and therapeutic targets for EC.

## Figures and Tables

**Table 1:** Regression coefficients of the seven PTEN mutational status-associated prognostic genes.

Id	Coef	HR	HR.95 L	HR.95H	*P* value
GDPD2	0.202281	1.224192	1.0066	1.48882	0.042781
GRB7	0.002907	1.002911	1.000964	1.004862	0.003377
KCNK9	0.287543	1.333148	1.113335	1.596361	0.001761
MUC3A	0.124442	1.132517	1.051765	1.219468	0.000976
MYT1	0.449528	1.567573	1.155313	2.126944	0.003887
RPS6KA6	0.265031	1.303471	0.941477	1.804652	0.110342
TSPYL5	0.035858	1.036509	1.013534	1.060005	0.001716

## Data Availability

All data generated or analyzed during this study are included in this published article.

## Supplementary Materials

Supplementary Materials Supplementary Figure 1: validation of a nomogram model in the clinical cohort. (A) A nomogram for predicting the 1-, 3-, and 5-year overall survival rates of EC patients. (B–D) The calibration curve at 1, 3, and 5 years. (E) A DCA curve was used to evaluate the accuracy of the nomogram model. Supplementary Table 1: the sequences of primers used for RT-qPCR. Supplementary Table 2: two hundred and twenty-four DEGs (37 upregulated genes and 187 downregulated genes) between the EC patients with PTEN mutation or not. Supplementary Table 3: eighty-four DEGs with prognostic value were selected by univariate Cox regression analysis.
