# Supplementary material for: Significance of a PTEN Mutational Status-Associated Gene Signature in the Progression and Prognosis of Endometrial Carcinoma
Source: Oxid Med Cell Longev. 2022 Feb 23;2022:5130648. doi: 10.1155/2022/5130648 (PMC8890874; doi:10.1155/2022/5130648)
Supplement: Supplementary Materials — Supplementary Figure 1: validation of a nomogram model in the clinical cohort. (A) A nomogram for predicting the 1-, 3-, and 5-year overall survival rates of EC patients. (B–D) The calibration curve at 1, 3, and 5 years. (E) A DCA curve was used to evaluate the accuracy of the nomogram model. Supplementary Table 1: the sequences of primers used for RT-qPCR. Supplementary Table 2: two hundred and twenty-four DEGs (37 upregulated genes and 187 downregulated genes) between the EC patients with PTEN mutation or not. Supplementary Table 3: eighty-four DEGs with prognostic value were selected by univariate Cox regression analysis. [file 5130648.f1.zip › Supplementary table 1 (1).docx]

**Supplementary Table 1** The sequences of primers used for RT-qPCR.

| Gene | Sequences of the primers |
| --- | --- |
| GDPD2 | F:5' TCTTCCTCACCTTCCTCCTTTCCC 3'  R:5’ CTATGCCACTCCTGCTGCCATTG 3’ |
| GRB7 | F:5' TAGACGGAGCCAGACTTCGGAAC 3’  R:5’ CGGTGGTTGGGATGAGGAGAGG 3’ |
| KCNK9 | F:5' TCACCTGCTCATACTCGCTCTCC 3’  R:5’ GCCGCTCATCCTCACTGTTCATG 3’ |
| MUC3A | F:5' GCTCCAGACCAGATGCCAGAATG 3’  R:5’ TCACCTGCTCATACTCGCTCTCC 3’ |
| MYT1 | F:5’ GATCCTTCCAAGAGTAGCTCCAATTCC 3’  R:5' CTGTGGCTTCGTGCTGAGGTTC 3’ |
| RPS6KA6 | F:5’ GAGCACCTCACAGCAATACTCCATC 3’  R:5' ATACTCTGACCCTGGGACACACTG 3’ |
| TSPYL5 | F:5' CGACGGAAGGCAGCATGGATAC3'  R:5’ CACTGGATTGGAGTAGAACGAGACAC 3’ |
| GAPDH | F:5’ CAAGGCTGTGGGCAAGGTCATC 3’  R:5’ GTGTCGCTGTTGAAGTCAGAGGAG 3’ |
