# Supplementary material for: Significance of a PTEN Mutational Status-Associated Gene Signature in the Progression and Prognosis of Endometrial Carcinoma
Source: Oxid Med Cell Longev. 2022 Feb 23;2022:5130648. doi: 10.1155/2022/5130648 (PMC8890874; doi:10.1155/2022/5130648)
Supplement: Supplementary Materials — Supplementary Figure 1: validation of a nomogram model in the clinical cohort. (A) A nomogram for predicting the 1-, 3-, and 5-year overall survival rates of EC patients. (B–D) The calibration curve at 1, 3, and 5 years. (E) A DCA curve was used to evaluate the accuracy of the nomogram model. Supplementary Table 1: the sequences of primers used for RT-qPCR. Supplementary Table 2: two hundred and twenty-four DEGs (37 upregulated genes and 187 downregulated genes) between the EC patients with PTEN mutation or not. Supplementary Table 3: eighty-four DEGs with prognostic value were selected by univariate Cox regression analysis. [file 5130648.f1.zip › Supplementary table 2 (1).docx]

**Supplementary Table 2** Two hundred and twenty-four DEGs (37 upregulated genes and 187 downregulated genes) between the EC patients with PTEN mutation or not.

| gene | conMean | treatMean | logFC | pValue | fdr |
| --- | --- | --- | --- | --- | --- |
| TM4SF20 | 0.772231 | 0.014727 | -5.71251 | 0.016783 | 0.026184 |
| PAGE5 | 1.312832 | 0.04423 | -4.89151 | 0.000527 | 0.001142 |
| PAGE2B | 3.024796 | 0.122874 | -4.62158 | 2.02E-09 | 1.18E-08 |
| SOX11 | 1.625333 | 0.066438 | -4.61257 | 1.70E-16 | 3.49E-15 |
| ERAS | 3.269834 | 0.1518 | -4.42898 | 4.54E-10 | 2.99E-09 |
| SLC7A10 | 0.841318 | 0.04136 | -4.34634 | 6.53E-29 | 1.67E-26 |
| TNMD | 1.00848 | 0.053047 | -4.24877 | 0.00024 | 0.000558 |
| UCMA | 2.02144 | 0.108901 | -4.21429 | 0.04535 | 0.06389 |
| CLDN6 | 67.72804 | 3.724256 | -4.18473 | 1.80E-33 | 2.02E-30 |
| EYA4 | 0.873207 | 0.050133 | -4.12248 | 5.61E-26 | 7.72E-24 |
| XAGE2B | 3.528538 | 0.207795 | -4.08584 | 1.98E-31 | 1.03E-28 |
| DLK1 | 4.102316 | 0.254143 | -4.01273 | 4.44E-12 | 4.14E-11 |
| L1CAM | 16.08561 | 1.012271 | -3.9901 | 2.06E-35 | 3.48E-32 |
| SLC6A15 | 0.638516 | 0.042784 | -3.89958 | 6.07E-28 | 1.17E-25 |
| NR0B1 | 0.872957 | 0.060573 | -3.84916 | 5.24E-13 | 5.71E-12 |
| GAL3ST3 | 3.817292 | 0.270815 | -3.81717 | 8.26E-24 | 6.56E-22 |
| VSTM2B | 0.706064 | 0.050756 | -3.79816 | 5.10E-17 | 1.17E-15 |
| IGFBP1 | 5.1566 | 0.370784 | -3.79777 | 0.00443 | 0.007933 |
| CTCFL | 2.289084 | 0.165133 | -3.79307 | 5.94E-30 | 1.96E-27 |
| KNG1 | 0.529846 | 0.03995 | -3.72931 | 1.95E-06 | 6.76E-06 |
| LECT1 | 0.771728 | 0.061393 | -3.65195 | 3.25E-06 | 1.09E-05 |
| FLG | 0.61583 | 0.049116 | -3.64827 | 1.35E-14 | 1.98E-13 |
| BTBD17 | 1.343761 | 0.107778 | -3.64014 | 6.46E-20 | 2.55E-18 |
| SLC6A11 | 0.537206 | 0.043109 | -3.6394 | 1.12E-35 | 2.17E-32 |
| PNOC | 10.01973 | 0.808649 | -3.63119 | 1.93E-20 | 8.60E-19 |
| CARTPT | 1.588923 | 0.1325 | -3.58398 | 0.000163 | 0.000391 |
| HIF3A | 3.641197 | 0.305891 | -3.57332 | 4.50E-38 | 2.18E-34 |
| CALCB | 6.375179 | 0.538504 | -3.56544 | 2.32E-09 | 1.35E-08 |
| UPK2 | 11.71934 | 0.995946 | -3.55668 | 8.26E-08 | 3.60E-07 |
| AHSG | 1.081825 | 0.092312 | -3.5508 | 0.002026 | 0.003903 |
| KLK5 | 12.22771 | 1.055371 | -3.53433 | 4.61E-12 | 4.29E-11 |
| CRTAC1 | 4.663986 | 0.40417 | -3.52853 | 2.61E-29 | 7.48E-27 |
| KLK6 | 36.78292 | 3.276221 | -3.48893 | 2.00E-16 | 4.01E-15 |
| MAGEA10 | 1.367221 | 0.124632 | -3.4555 | 7.57E-16 | 1.36E-14 |
| ACTL8 | 5.835733 | 0.57097 | -3.35343 | 2.00E-17 | 4.96E-16 |
| LHX1 | 2.786973 | 0.275394 | -3.33913 | 2.30E-11 | 1.89E-10 |
| GFAP | 1.71005 | 0.171459 | -3.3181 | 2.27E-14 | 3.20E-13 |
| IGF2 | 447.4267 | 47.68556 | -3.23003 | 6.43E-08 | 2.86E-07 |
| TDRD12 | 1.418978 | 0.154594 | -3.1983 | 3.28E-18 | 9.75E-17 |
| SST | 167.5366 | 18.49946 | -3.17892 | 1.04E-21 | 5.73E-20 |
| LBP | 17.50517 | 1.968987 | -3.15226 | 0.001515 | 0.00299 |
| TM4SF5 | 0.705042 | 0.079772 | -3.14375 | 3.81E-05 | 0.000104 |
| GFRA1 | 2.436843 | 0.27776 | -3.1331 | 2.01E-07 | 8.23E-07 |
| LYPD2 | 26.34504 | 3.094216 | -3.08989 | 3.36E-07 | 1.33E-06 |
| MYOD1 | 0.657612 | 0.077855 | -3.07838 | 4.48E-14 | 6.06E-13 |
| KLK7 | 16.76738 | 2.014714 | -3.05701 | 6.47E-22 | 3.80E-20 |
| EEF1A2 | 47.29178 | 5.742254 | -3.0419 | 4.89E-09 | 2.69E-08 |
| MAGEC2 | 2.490566 | 0.307559 | -3.01754 | 4.28E-19 | 1.45E-17 |
| ZNF334 | 1.07975 | 0.134209 | -3.00814 | 4.00E-26 | 5.75E-24 |
| CLDN19 | 1.00769 | 0.126146 | -2.99788 | 1.98E-19 | 7.09E-18 |
| PVALB | 1.554435 | 0.201915 | -2.94457 | 4.52E-11 | 3.58E-10 |
| BRINP1 | 0.677112 | 0.088591 | -2.93416 | 6.77E-17 | 1.51E-15 |
| MAG | 1.082551 | 0.141895 | -2.93154 | 2.08E-24 | 1.89E-22 |
| MAGEA11 | 2.429045 | 0.3185 | -2.93102 | 7.46E-13 | 7.93E-12 |
| BPIFB4 | 0.735023 | 0.097548 | -2.9136 | 0.019324 | 0.029733 |
| TSHR | 0.577061 | 0.076965 | -2.90644 | 9.33E-10 | 5.78E-09 |
| RSPO4 | 3.888883 | 0.524576 | -2.89013 | 5.21E-40 | 7.03E-36 |
| NPY | 2.852574 | 0.388623 | -2.87582 | 1.37E-17 | 3.50E-16 |
| SSTR1 | 1.39995 | 0.192256 | -2.86427 | 7.61E-13 | 8.06E-12 |
| ADAMTS16 | 1.63803 | 0.230728 | -2.82769 | 1.06E-12 | 1.10E-11 |
| PAGE2 | 7.026696 | 0.997341 | -2.81669 | 5.58E-12 | 5.10E-11 |
| SLC6A13 | 0.927725 | 0.132825 | -2.80417 | 1.39E-24 | 1.34E-22 |
| COL9A1 | 8.355739 | 1.198111 | -2.80201 | 1.63E-13 | 1.95E-12 |
| PLAC1 | 0.794999 | 0.114893 | -2.79066 | 1.11E-10 | 8.25E-10 |
| FXYD7 | 0.720734 | 0.105714 | -2.76931 | 3.15E-18 | 9.39E-17 |
| COMP | 25.71089 | 3.804269 | -2.75669 | 3.62E-09 | 2.03E-08 |
| WT1 | 8.856007 | 1.337684 | -2.72692 | 3.06E-10 | 2.08E-09 |
| KCNK2 | 0.806581 | 0.124289 | -2.69812 | 1.63E-13 | 1.95E-12 |
| CRB2 | 1.443189 | 0.222679 | -2.69622 | 2.02E-14 | 2.86E-13 |
| PAPPA2 | 1.014874 | 0.156678 | -2.69542 | 0.007281 | 0.012363 |
| MYT1 | 0.451005 | 0.07058 | -2.6758 | 2.07E-33 | 2.11E-30 |
| PPP2R2B | 0.600897 | 0.094255 | -2.67248 | 1.53E-14 | 2.23E-13 |
| TCEAL5 | 3.156787 | 0.495225 | -2.6723 | 1.06E-15 | 1.88E-14 |
| GCGR | 1.684832 | 0.265889 | -2.66371 | 4.71E-12 | 4.37E-11 |
| KLK8 | 9.436724 | 1.49525 | -2.6579 | 5.63E-20 | 2.28E-18 |
| APOA1 | 30.78884 | 4.904082 | -2.65035 | 3.08E-08 | 1.46E-07 |
| ZNF229 | 0.707958 | 0.113204 | -2.64473 | 3.96E-16 | 7.49E-15 |
| DLGAP3 | 2.287587 | 0.372488 | -2.61856 | 6.86E-28 | 1.30E-25 |
| WNT7A | 33.00346 | 5.431013 | -2.60332 | 1.59E-22 | 1.02E-20 |
| MCCD1 | 1.833514 | 0.319864 | -2.51908 | 1.03E-09 | 6.33E-09 |
| PTX3 | 2.901579 | 0.51253 | -2.50113 | 3.90E-25 | 4.50E-23 |
| DOK5 | 3.105782 | 0.557266 | -2.47852 | 6.81E-20 | 2.68E-18 |
| KRTAP2-3 | 2.026018 | 0.367279 | -2.4637 | 5.83E-14 | 7.67E-13 |
| DUSP9 | 2.507975 | 0.456043 | -2.45928 | 2.23E-22 | 1.41E-20 |
| GPRIN2 | 2.637578 | 0.480857 | -2.45553 | 5.95E-36 | 1.34E-32 |
| NKAIN4 | 7.290519 | 1.330935 | -2.45358 | 5.34E-21 | 2.56E-19 |
| LRCH2 | 1.327656 | 0.243667 | -2.4459 | 1.19E-16 | 2.52E-15 |
| FOXI3 | 0.795581 | 0.146086 | -2.4452 | 3.07E-20 | 1.30E-18 |
| KCNQ2 | 1.121914 | 0.206412 | -2.44236 | 1.95E-05 | 5.65E-05 |
| DCAF12L1 | 2.136936 | 0.393567 | -2.44086 | 4.49E-21 | 2.18E-19 |
| PRSS50 | 4.756237 | 0.877322 | -2.43864 | 2.55E-22 | 1.60E-20 |
| CNTFR | 6.29442 | 1.169423 | -2.42828 | 4.11E-15 | 6.54E-14 |
| IGLON5 | 2.912587 | 0.5445 | -2.4193 | 1.01E-10 | 7.52E-10 |
| COL4A3 | 0.558413 | 0.104672 | -2.41545 | 2.37E-15 | 3.97E-14 |
| HOXD1 | 1.432268 | 0.269409 | -2.41043 | 3.93E-08 | 1.83E-07 |
| SLITRK2 | 0.642824 | 0.12121 | -2.40692 | 4.26E-10 | 2.82E-09 |
| HOXA4 | 2.700216 | 0.509378 | -2.40627 | 1.27E-17 | 3.29E-16 |
| COX8C | 1.046882 | 0.197566 | -2.40569 | 0.002183 | 0.004175 |
| PCDH10 | 1.182875 | 0.223545 | -2.40366 | 2.40E-08 | 1.15E-07 |
| AOC1 | 81.28517 | 15.63572 | -2.37815 | 2.25E-10 | 1.58E-09 |
| PPARGC1A | 0.766397 | 0.147736 | -2.37507 | 2.01E-19 | 7.21E-18 |
| ADRA1B | 2.417704 | 0.466193 | -2.37464 | 2.33E-21 | 1.19E-19 |
| ZNF311 | 0.94773 | 0.183637 | -2.36762 | 1.39E-29 | 4.09E-27 |
| FOXD3 | 0.451967 | 0.087622 | -2.36685 | 2.61E-11 | 2.13E-10 |
| FOLR3 | 1.568691 | 0.304494 | -2.36508 | 7.52E-16 | 1.35E-14 |
| COL4A4 | 1.154552 | 0.224149 | -2.3648 | 2.14E-16 | 4.25E-15 |
| TRHDE | 0.698235 | 0.135979 | -2.36033 | 9.85E-16 | 1.75E-14 |
| HBE1 | 0.462788 | 0.09101 | -2.34626 | 0.000442 | 0.00097 |
| COL22A1 | 1.580508 | 0.311148 | -2.34472 | 3.94E-13 | 4.41E-12 |
| MYO7B | 1.59835 | 0.31534 | -2.3416 | 1.52E-15 | 2.61E-14 |
| MAFA | 0.890234 | 0.176187 | -2.33708 | 2.85E-15 | 4.65E-14 |
| RNF212 | 2.560887 | 0.507477 | -2.33523 | 4.10E-25 | 4.65E-23 |
| COX6A2 | 0.769577 | 0.152571 | -2.33459 | 2.32E-17 | 5.70E-16 |
| VTN | 0.737032 | 0.146592 | -2.32992 | 0.019767 | 0.030325 |
| ALK | 0.445453 | 0.089098 | -2.32181 | 9.21E-26 | 1.21E-23 |
| ELFN2 | 0.43403 | 0.087247 | -2.31461 | 7.20E-18 | 2.01E-16 |
| SGCG | 0.67581 | 0.136082 | -2.31214 | 1.39E-14 | 2.04E-13 |
| AARD | 1.649433 | 0.335368 | -2.29815 | 2.42E-27 | 4.24E-25 |
| MEOX1 | 4.492207 | 0.921722 | -2.28502 | 1.30E-10 | 9.47E-10 |
| FXYD2 | 0.98426 | 0.202432 | -2.2816 | 8.07E-13 | 8.52E-12 |
| CALHM3 | 0.900523 | 0.185424 | -2.27993 | 1.83E-11 | 1.52E-10 |
| SLC12A3 | 1.142858 | 0.235565 | -2.27845 | 0.001155 | 0.002339 |
| KCNJ16 | 1.704044 | 0.353922 | -2.26746 | 1.79E-07 | 7.39E-07 |
| SYT13 | 4.226917 | 0.887959 | -2.25104 | 4.84E-38 | 2.18E-34 |
| NKAIN2 | 0.426519 | 0.089616 | -2.25078 | 0.001836 | 0.003565 |
| CLDN9 | 11.99967 | 2.53778 | -2.24136 | 6.32E-30 | 1.99E-27 |
| PLAG1 | 1.271749 | 0.269425 | -2.23886 | 6.69E-29 | 1.67E-26 |
| RHOXF1 | 2.397229 | 0.509905 | -2.23307 | 0.00203 | 0.003909 |
| TNNT3 | 1.534296 | 0.327612 | -2.22752 | 2.55E-05 | 7.22E-05 |
| GDPD2 | 0.439642 | 0.094105 | -2.22398 | 1.54E-16 | 3.18E-15 |
| AVPR2 | 1.679446 | 0.359651 | -2.22331 | 2.35E-14 | 3.30E-13 |
| GRB7 | 44.18649 | 9.466635 | -2.22268 | 7.53E-29 | 1.84E-26 |
| CLDN16 | 4.603423 | 0.986966 | -2.22164 | 1.04E-20 | 4.86E-19 |
| SLC39A5 | 1.64199 | 0.352837 | -2.21837 | 1.64E-12 | 1.64E-11 |
| DOCK3 | 0.651172 | 0.140265 | -2.21488 | 1.28E-25 | 1.64E-23 |
| SELV | 0.802584 | 0.172942 | -2.21436 | 1.12E-09 | 6.89E-09 |
| FBXO17 | 5.191105 | 1.11969 | -2.21294 | 5.17E-22 | 3.09E-20 |
| PNMA3 | 5.504441 | 1.188387 | -2.21159 | 2.63E-35 | 3.95E-32 |
| IQSEC3 | 0.936641 | 0.202309 | -2.21094 | 1.16E-19 | 4.41E-18 |
| RPS6KA6 | 0.554307 | 0.120239 | -2.20478 | 3.84E-20 | 1.59E-18 |
| GRIK5 | 4.166109 | 0.907106 | -2.19936 | 1.58E-20 | 7.12E-19 |
| CDH6 | 8.42983 | 1.840266 | -2.19559 | 2.57E-20 | 1.11E-18 |
| MAGEA4 | 4.601343 | 1.008808 | -2.1894 | 3.60E-16 | 6.85E-15 |
| ZDBF2 | 0.761623 | 0.168079 | -2.17994 | 9.51E-13 | 9.90E-12 |
| CD22 | 1.950091 | 0.435161 | -2.16392 | 1.59E-15 | 2.72E-14 |
| SYT14 | 0.58223 | 0.130043 | -2.1626 | 3.94E-16 | 7.46E-15 |
| KCNK9 | 0.601792 | 0.134415 | -2.16257 | 9.75E-27 | 1.57E-24 |
| MUC3A | 0.456523 | 0.10239 | -2.15661 | 3.23E-05 | 8.94E-05 |
| NTSR1 | 0.466799 | 0.104929 | -2.15339 | 2.59E-13 | 3.00E-12 |
| RYR1 | 1.295873 | 0.29138 | -2.15295 | 8.12E-28 | 1.52E-25 |
| KCNJ4 | 1.518397 | 0.343551 | -2.14395 | 2.99E-13 | 3.43E-12 |
| C19orf84 | 0.998066 | 0.22636 | -2.14052 | 1.28E-11 | 1.09E-10 |
| SLC6A12 | 4.133776 | 0.940472 | -2.136 | 2.94E-21 | 1.48E-19 |
| TNNT1 | 31.84735 | 7.268229 | -2.1315 | 1.02E-22 | 6.85E-21 |
| TRO | 5.375371 | 1.245377 | -2.10978 | 1.92E-31 | 1.03E-28 |
| KCNE5 | 0.909726 | 0.213106 | -2.09386 | 0.002102 | 0.004036 |
| CBLN2 | 0.527827 | 0.123987 | -2.08987 | 0.003269 | 0.006007 |
| KCNS1 | 0.964389 | 0.227239 | -2.08541 | 5.78E-25 | 6.34E-23 |
| IRS2 | 5.39405 | 1.272057 | -2.08421 | 3.30E-29 | 8.82E-27 |
| BMP3 | 2.491778 | 0.58781 | -2.08375 | 0.000789 | 0.00165 |
| DAB1 | 0.475986 | 0.112556 | -2.08027 | 5.47E-10 | 3.54E-09 |
| CDKN2A | 28.86083 | 6.826358 | -2.07992 | 2.62E-31 | 1.22E-28 |
| RNF182 | 2.34002 | 0.554065 | -2.07839 | 2.66E-25 | 3.13E-23 |
| ANKLE1 | 2.465788 | 0.584201 | -2.07751 | 3.61E-21 | 1.79E-19 |
| TUBB4A | 5.75574 | 1.370047 | -2.07078 | 2.85E-23 | 2.10E-21 |
| VCX3A | 0.67733 | 0.161243 | -2.07063 | 7.44E-07 | 2.79E-06 |
| VGLL1 | 6.728796 | 1.602248 | -2.07025 | 8.85E-18 | 2.38E-16 |
| BCAM | 258.3117 | 61.5868 | -2.06842 | 1.46E-31 | 8.20E-29 |
| IGF2BP1 | 1.043654 | 0.249198 | -2.06628 | 4.23E-24 | 3.53E-22 |
| JPH3 | 0.819223 | 0.195917 | -2.06401 | 5.30E-07 | 2.03E-06 |
| KCNIP1 | 0.933265 | 0.224979 | -2.0525 | 1.05E-05 | 3.19E-05 |
| SNCG | 23.74384 | 5.724266 | -2.05239 | 3.47E-11 | 2.79E-10 |
| GABRQ | 0.518172 | 0.125228 | -2.04887 | 1.12E-24 | 1.12E-22 |
| BARX2 | 1.683082 | 0.407402 | -2.04658 | 1.08E-12 | 1.11E-11 |
| MYLPF | 3.75533 | 0.910341 | -2.04446 | 1.33E-13 | 1.62E-12 |
| IGF2BP2 | 18.28461 | 4.454918 | -2.03716 | 6.96E-32 | 4.48E-29 |
| PCSK1N | 41.15395 | 10.03713 | -2.03568 | 2.25E-23 | 1.70E-21 |
| COX4I2 | 6.388456 | 1.56266 | -2.03146 | 2.42E-07 | 9.76E-07 |
| ENHO | 3.722678 | 0.913161 | -2.0274 | 8.80E-17 | 1.92E-15 |
| SPON1 | 79.30738 | 19.46415 | -2.02664 | 1.35E-17 | 3.47E-16 |
| NUDT11 | 1.62743 | 0.399918 | -2.02482 | 1.10E-08 | 5.65E-08 |
| ADRA2B | 0.928218 | 0.228946 | -2.01946 | 1.64E-15 | 2.80E-14 |
| TSPYL5 | 4.758654 | 1.174186 | -2.01889 | 0.000421 | 0.000929 |
| RSPO3 | 5.296936 | 1.30719 | -2.01869 | 1.88E-05 | 5.46E-05 |
| NLRP7 | 0.861047 | 0.213556 | -2.01147 | 4.07E-08 | 1.89E-07 |
| PDE6G | 2.978566 | 0.74099 | -2.00709 | 4.68E-15 | 7.38E-14 |
| HMX1 | 0.738756 | 0.184689 | -2 | 6.38E-05 | 0.000166 |
| WDR38 | 4.122977 | 16.49438 | 2.000216 | 7.11E-12 | 6.42E-11 |
| C1orf194 | 6.423039 | 25.864 | 2.009617 | 1.67E-19 | 6.11E-18 |
| LRRC18 | 0.359523 | 1.477511 | 2.039015 | 4.15E-16 | 7.79E-15 |
| IHH | 10.33956 | 42.60936 | 2.042996 | 4.46E-28 | 9.09E-26 |
| MMP26 | 2.999541 | 12.44574 | 2.052838 | 2.84E-11 | 2.30E-10 |
| FAM92B | 1.761215 | 7.343462 | 2.059889 | 3.51E-12 | 3.34E-11 |
| OVGP1 | 18.65546 | 79.01614 | 2.08255 | 1.02E-11 | 8.94E-11 |
| LPPR3 | 0.756123 | 3.218844 | 2.08985 | 4.70E-12 | 4.36E-11 |
| MS4A8 | 4.535228 | 19.34347 | 2.092599 | 4.00E-05 | 0.000109 |
| CNGA4 | 0.647535 | 2.766678 | 2.095126 | 1.95E-14 | 2.79E-13 |
| GAS2L2 | 0.85559 | 3.759767 | 2.135651 | 2.51E-17 | 6.11E-16 |
| TEKT4 | 0.84517 | 3.726752 | 2.140606 | 1.04E-14 | 1.55E-13 |
| ENSG00000188396 | 0.631274 | 2.899498 | 2.199466 | 2.87E-14 | 3.99E-13 |
| AZU1 | 0.216131 | 1.005391 | 2.217776 | 9.54E-16 | 1.69E-14 |
| OMG | 1.046531 | 4.924881 | 2.234474 | 4.03E-17 | 9.42E-16 |
| SCGB1D1 | 6.599175 | 31.39795 | 2.250313 | 1.50E-06 | 5.32E-06 |
| MUC6 | 3.129504 | 15.05856 | 2.266578 | 6.86E-06 | 2.17E-05 |
| SCGB1D2 | 66.03498 | 319.1001 | 2.272706 | 1.89E-14 | 2.71E-13 |
| CCDC60 | 0.341936 | 1.660984 | 2.280238 | 1.65E-12 | 1.65E-11 |
| SCGB1A1 | 5.658911 | 27.68972 | 2.290754 | 7.67E-11 | 5.86E-10 |
| HPR | 0.120277 | 0.592169 | 2.299646 | 0.000322 | 0.000727 |
| DEFB4A | 0.39868 | 2.040165 | 2.355382 | 1.96E-14 | 2.79E-13 |
| LRRC26 | 1.268932 | 6.770164 | 2.415576 | 4.51E-28 | 9.09E-26 |
| TMEM210 | 0.085857 | 0.46031 | 2.422593 | 2.73E-28 | 5.85E-26 |
| PZP | 0.279501 | 1.507476 | 2.431208 | 1.37E-09 | 8.27E-09 |
| MUC5B | 5.838796 | 31.71538 | 2.44144 | 6.33E-13 | 6.77E-12 |
| TFF3 | 120.5667 | 760.6299 | 2.657363 | 2.92E-34 | 3.58E-31 |
| MUC5AC | 0.519692 | 3.31305 | 2.67243 | 0.008922 | 0.01483 |
| MUC7 | 0.098886 | 0.647399 | 2.710823 | 1.25E-12 | 1.27E-11 |
| L1TD1 | 0.052362 | 0.351665 | 2.747609 | 0.008609 | 0.014364 |
| PIP | 0.578022 | 3.931945 | 2.766047 | 1.02E-11 | 8.88E-11 |
| GLYATL3 | 0.157524 | 1.364415 | 3.114637 | 1.74E-10 | 1.25E-09 |
| PMCH | 0.143437 | 1.273497 | 3.150306 | 0.00489 | 0.008663 |
| BPIFB2 | 0.660209 | 6.091304 | 3.205757 | 7.12E-11 | 5.48E-10 |
| GP2 | 0.342753 | 4.196527 | 3.613953 | 5.10E-07 | 1.96E-06 |
| SCGB3A1 | 13.0987 | 168.9225 | 3.688865 | 1.07E-10 | 7.98E-10 |
| SCGB2A2 | 2.452017 | 55.64647 | 4.504249 | 9.15E-24 | 7.22E-22 |
