# Supplementary material for: Significance of a PTEN Mutational Status-Associated Gene Signature in the Progression and Prognosis of Endometrial Carcinoma
Source: Oxid Med Cell Longev. 2022 Feb 23;2022:5130648. doi: 10.1155/2022/5130648 (PMC8890874; doi:10.1155/2022/5130648)
Supplement: Supplementary Materials — Supplementary Figure 1: validation of a nomogram model in the clinical cohort. (A) A nomogram for predicting the 1-, 3-, and 5-year overall survival rates of EC patients. (B–D) The calibration curve at 1, 3, and 5 years. (E) A DCA curve was used to evaluate the accuracy of the nomogram model. Supplementary Table 1: the sequences of primers used for RT-qPCR. Supplementary Table 2: two hundred and twenty-four DEGs (37 upregulated genes and 187 downregulated genes) between the EC patients with PTEN mutation or not. Supplementary Table 3: eighty-four DEGs with prognostic value were selected by univariate Cox regression analysis. [file 5130648.f1.zip › Supplementary table 3 (1).docx]

**Supplementary Table 3** Eighty-four DEGs with prognostic value were selected by univariate Cox regression analysis.

| id | HR | HR.95L | HR.95H | pvalue |
| --- | --- | --- | --- | --- |
| KCNK9 | 1.447147 | 1.264264 | 1.656484 | 8.24E-08 |
| MYT1 | 1.897098 | 1.479091 | 2.433238 | 4.60E-07 |
| DOK5 | 1.062413 | 1.036868 | 1.088589 | 1.09E-06 |
| TM4SF20 | 1.046298 | 1.026261 | 1.066726 | 4.49E-06 |
| RSPO4 | 1.084555 | 1.04726 | 1.123179 | 5.46E-06 |
| ADAMTS16 | 1.116223 | 1.064425 | 1.170543 | 5.75E-06 |
| MUC3A | 1.169826 | 1.09185 | 1.253372 | 8.32E-06 |
| RPS6KA6 | 1.737839 | 1.36229 | 2.216919 | 8.64E-06 |
| TSPYL5 | 1.045406 | 1.024819 | 1.066406 | 1.21E-05 |
| PAGE5 | 1.033369 | 1.017889 | 1.049083 | 2.02E-05 |
| GDPD2 | 1.436483 | 1.207653 | 1.708672 | 4.29E-05 |
| HIF3A | 1.050787 | 1.025044 | 1.077176 | 9.06E-05 |
| SLC6A11 | 1.647321 | 1.281685 | 2.117264 | 9.70E-05 |
| MEOX1 | 1.029987 | 1.014346 | 1.045869 | 0.000154 |
| L1CAM | 1.014961 | 1.007131 | 1.022853 | 0.000171 |
| GFAP | 1.023221 | 1.010861 | 1.035731 | 0.000214 |
| GRB7 | 1.003083 | 1.001419 | 1.004751 | 0.00028 |
| DCAF12L1 | 1.146945 | 1.064329 | 1.235974 | 0.000325 |
| SLITRK2 | 1.174 | 1.07405 | 1.28325 | 0.00041 |
| KRTAP2-3 | 1.041784 | 1.016924 | 1.067251 | 0.000894 |
| BARX2 | 1.095479 | 1.03796 | 1.156185 | 0.00092 |
| MCCD1 | 1.055016 | 1.022045 | 1.089051 | 0.000946 |
| COX4I2 | 1.012773 | 1.005177 | 1.020426 | 0.000952 |
| BCAM | 1.001169 | 1.000463 | 1.001876 | 0.001175 |
| PPP2R2B | 1.243589 | 1.08758 | 1.421977 | 0.001435 |
| TRO | 1.065428 | 1.0244 | 1.108098 | 0.00156 |
| GPRIN2 | 1.110346 | 1.039339 | 1.186205 | 0.001907 |
| SCGB2A2 | 1.000373 | 1.000137 | 1.000609 | 0.001919 |
| PNMA3 | 1.046008 | 1.01558 | 1.077348 | 0.002823 |
| TM4SF5 | 1.05714 | 1.019178 | 1.096516 | 0.002901 |
| VGLL1 | 1.027441 | 1.009248 | 1.045962 | 0.00298 |
| TSHR | 1.137197 | 1.04432 | 1.238334 | 0.003101 |
| SCGB1A1 | 1.001409 | 1.000466 | 1.002353 | 0.003411 |
| BPIFB4 | 1.044807 | 1.014456 | 1.076065 | 0.003566 |
| PNOC | 1.01266 | 1.004113 | 1.021279 | 0.003623 |
| TUBB4A | 1.03347 | 1.010548 | 1.056911 | 0.004016 |
| DLGAP3 | 1.057014 | 1.017819 | 1.097719 | 0.004026 |
| PIP | 1.00531 | 1.001688 | 1.008945 | 0.004031 |
| GAL3ST3 | 1.042901 | 1.013329 | 1.073335 | 0.004207 |
| MUC7 | 1.026285 | 1.007906 | 1.044999 | 0.004893 |
| IHH | 0.990342 | 0.983643 | 0.997087 | 0.005072 |
| HBE1 | 1.15631 | 1.044076 | 1.280609 | 0.005304 |
| DOCK3 | 1.500969 | 1.127034 | 1.99897 | 0.005469 |
| HOXD1 | 1.085307 | 1.024153 | 1.150113 | 0.005666 |
| IGF2BP2 | 1.017335 | 1.004726 | 1.030101 | 0.006912 |
| AARD | 1.0199 | 1.005387 | 1.034621 | 0.007044 |
| NTSR1 | 1.198427 | 1.050344 | 1.367388 | 0.007148 |
| PLAG1 | 1.218826 | 1.054894 | 1.408233 | 0.007251 |
| WNT7A | 1.005431 | 1.001429 | 1.009448 | 0.007765 |
| CLDN9 | 1.008193 | 1.002138 | 1.014286 | 0.00794 |
| CDH6 | 1.019833 | 1.005109 | 1.034773 | 0.008128 |
| CLDN16 | 1.01714 | 1.004281 | 1.030164 | 0.008845 |
| JPH3 | 1.108046 | 1.025269 | 1.197507 | 0.0096 |
| LHX1 | 1.036459 | 1.008429 | 1.065269 | 0.010467 |
| TEKT4 | 0.886018 | 0.807285 | 0.97243 | 0.01081 |
| GABRQ | 1.368149 | 1.073684 | 1.743373 | 0.011248 |
| RSPO3 | 1.021502 | 1.004681 | 1.038604 | 0.012029 |
| PDE6G | 1.058393 | 1.011976 | 1.10694 | 0.013129 |
| SLC39A5 | 1.053188 | 1.010605 | 1.097565 | 0.013858 |
| PCSK1N | 1.004558 | 1.000884 | 1.008247 | 0.015001 |
| COL22A1 | 1.109217 | 1.020175 | 1.206031 | 0.015191 |
| ANKLE1 | 1.097302 | 1.018043 | 1.182732 | 0.015206 |
| SCGB1D1 | 1.000561 | 1.000098 | 1.001023 | 0.017473 |
| KLK6 | 1.00284 | 1.00048 | 1.005206 | 0.018318 |
| ENSG00000188396 | 0.862769 | 0.762525 | 0.976191 | 0.019163 |
| TDRD12 | 1.063454 | 1.009391 | 1.120413 | 0.020829 |
| FXYD2 | 1.081757 | 1.01146 | 1.15694 | 0.021885 |
| AZU1 | 0.677564 | 0.485075 | 0.946437 | 0.022441 |
| PPARGC1A | 1.162408 | 1.020701 | 1.323789 | 0.023277 |
| GAS2L2 | 0.913053 | 0.843666 | 0.988148 | 0.024093 |
| CLDN6 | 1.002576 | 1.000326 | 1.004832 | 0.024841 |
| NKAIN4 | 1.01395 | 1.001703 | 1.026346 | 0.025451 |
| CDKN2A | 1.00663 | 1.000798 | 1.012497 | 0.025818 |
| NR0B1 | 1.056953 | 1.006401 | 1.110045 | 0.02675 |
| MYO7B | 1.040892 | 1.004087 | 1.079046 | 0.029107 |
| AOC1 | 1.00094 | 1.000087 | 1.001794 | 0.030838 |
| MAGEA10 | 1.064692 | 1.005548 | 1.127314 | 0.031578 |
| CRB2 | 1.072189 | 1.004458 | 1.144487 | 0.036298 |
| C19orf84 | 1.122927 | 1.006585 | 1.252715 | 0.037748 |
| FOXD3 | 1.165268 | 1.00862 | 1.346246 | 0.037849 |
| SST | 1.00035 | 1.000017 | 1.000682 | 0.0391 |
| WDR38 | 0.9833 | 0.967692 | 0.99916 | 0.039126 |
| XAGE2B | 1.027666 | 1.001313 | 1.054712 | 0.039495 |
| ALK | 1.284803 | 1.006221 | 1.640512 | 0.044463 |
