# Supplementary material for: Significance of a PTEN Mutational Status-Associated Gene Signature in the Progression and Prognosis of Endometrial Carcinoma
Source: Oxid Med Cell Longev. 2022 Feb 23;2022:5130648. doi: 10.1155/2022/5130648 (PMC8890874; doi:10.1155/2022/5130648)
Supplement: Supplementary Materials — Supplementary Figure 1: validation of a nomogram model in the clinical cohort. (A) A nomogram for predicting the 1-, 3-, and 5-year overall survival rates of EC patients. (B–D) The calibration curve at 1, 3, and 5 years. (E) A DCA curve was used to evaluate the accuracy of the nomogram model. Supplementary Table 1: the sequences of primers used for RT-qPCR. Supplementary Table 2: two hundred and twenty-four DEGs (37 upregulated genes and 187 downregulated genes) between the EC patients with PTEN mutation or not. Supplementary Table 3: eighty-four DEGs with prognostic value were selected by univariate Cox regression analysis. [file 5130648.f1.zip › Untitled document.pdf]

Supplementary Table 1 The sequences of primers used for RT-qPCR.

Supplementary Table 2 Two hundred and twenty-four DEGs (37 upregulated genes and 187 downregulated genes) between the EC patients with PTEN mutation or not.

Supplementary Table 3 Eighty-four DEGs with prognostic value were selected by univariate Cox regression analysis.

Supplementary Figure 1. Validation of a nomogram model in the clinical cohort. **(A)** A nomogram for predicting the 1-, 3-, and 5-year overall survival rates of EC patients. **(B–D)** The calibration curve at 1, 3, and 5 years. **(E)** A DCA curve was used to evaluate the accuracy of the nomogram model.
